# Priming for tolerance and cohesion at replication forks

**DOI:** 10.1080/19491034.2016.1149663

**Published:** 2016-02-18

**Authors:** Dana Branzei, Barnabas Szakal

**Affiliations:** IFOM, the FIRC Institute of Molecular Oncology, Milan, Italy

**Keywords:** Chromosome replication, DNA damage tolerance, fork reversal recombination, repriming, sister chromatid cohesion

## Abstract

Genome duplication is coupled with DNA damage tolerance (DDT) and chromatin structural changes. Recently we reported that mutations in Primase subunits or factors that bridge Polα/Primase with the replicative helicase, Ctf4, caused abnormal usage of DDT pathways, negatively influenced sister chromatid cohesion (SCC), and associated with increased fork reversal.^1^ We also found that cohesin, which is paradigmatic for SCC, facilitates recombination-mediated DDT. However, only the recombination defects of cohesin, but not of cohesion-defective Polα/Primase/Ctf4 mutants, were rescued by artificial tethering of sister chromatids. Genetic tests and electron microscopy analysis of replication intermediates made us propose that management of single-stranded DNA forming proximal to the fork is a critical determinant of chromosome and replication fork structure, and influences DDT pathway choice. Here we discuss the implications of our findings for understanding DDT regulation and cohesion establishment during replication, and outline directions to rationalize the relationship between these chromosome metabolism processes.

Genome replication, required for the propagation of all living cells and organisms, is a prodigious task made possible by the complex and timely interplay between replication factors, DNA repair activities, and other DNA metabolism pathways that ensure correct chromosome structure establishment.^2^ DNA damage stalls replication forks causing exposure of single stranded (ss) DNA, which in turn triggers activation of DNA damage tolerance (DDT) pathways that promote damage-bypass. As ssDNA is chemically fragile, timely activation of DDT pathways is crucial to prevent deleterious formation of double strand breaks (DSBs), a leading source of genome instability. Thus, transient formation of ssDNA during replication is unavoidable, but DDT mechanisms prevent replication-associated fragility by their ability to mediate damage-bypass.

However, DDT pathways are not entirely error-free. Two modes of DDT have been described in all eukaryotic species.^3^ One mode utilizes specialized trans-lesion synthesis (TLS) polymerases, which can replicate across bulky DNA lesions, but can occasionally cause incorporation of mutations. The TLS mode is therefore said to be error-prone or mutagenic. The other mode utilizes recombination to switch templates (template switching), from the damaged strand to a homologous template, generally the newly synthesized sister chromatid. Template switching is in principle error-free.^3^

Judging from the different implications that the two DDT modes have on genome integrity, an interesting question is whether cells are able to preferentially use error-free pathways first, while postponing error-prone pathways as last resort options. Recent findings suggest that this may be indeed the case. Notably, template switching is favored at early times during replication over other available DDT pathways,^4,5^ whereas mutation rates are generally low at early replicating regions.^6,7^ The underlying mechanism behind this preference remains largely mysterious, but the chromatin status (euchromatin versus heterochromatin) and topological transitions involving DNA bending correlate with the ability of cells to efficiently engage in recombination-mediated DDT rather than mutagenesis early during replication.^5,8,9^ As epigenetic modifications and genome architecture are tightly linked to the replication status,^10^ one must query the timing and location of DDT activation in relation to the replication fork.

DNA lesions transiently stall the replisome, but this does not necessarily imply uncoupling between the replisome and the replicative helicase.^2^ For example, in case of lagging strand lesions, Okazaki fragment synthesis will ensure repriming downstream of the lesion, without any effect on fork movement. Several approaches specifically interrogated the timing of DDT events. Studies using conditional or S phase-specific depletion of key DDT components reached the conclusion that DDT does not need to be coupled to the fork, being fully operational even if restricted to G2/M ^11,12^ Electron microscopy studies of bypass recombination products revealed that template switching, normally favored in S phase, is initiated primarily on gaps behind replication forks.^13^ If DDT events occur prevalently postreplicatively, then uncoupling between the leading strand replisome and the replicative helicase must also be generally relieved by restart of DNA synthesis downstream the lesions. How may this this happen?

In a recent study in the lab, we examined the role of repriming in DDT and template switch events triggered by DNA damage.^1^ For this, we synchronously released cells in S phase and, as source of DNA lesions, we used methyl methanesulfonate (MMS), an alkylating agent that is expected to cause lesions to a similar extent on both strands. Because the enzymes required for primer formation/repriming (Polα/Primase) are essential for viability, we employed hypomorphic alleles and experimental conditions that do not affect the timing of bulk DNA synthesis. We found that Polα/Primase mutants caused a strong decrease in template switch intermediate formation. The observed decrease was far beyond the one expected if only DDT events initiated from lagging strand lesions were affected by the employed mutations in the Polα/Primase complex. The reduction in template switch intermediates also associated with an increase in mutagenic DDT, substantiating the notion of innate flexibilities within DDT. We next used another mutant to address if such repriming events need to be coupled with the replicative helicase movement. In the context of the replisome, Polα/Primase is tethered and functionally coupled to the replicative helicase mini-chromosome maintenance MCM by the conserved replisome architectural factor, Ctf4.^14^ Notably, this coupling is not essential for genome duplication and for repriming in *S. cerevisiae*. Strikingly, deletion of *CTF4* caused similar defects with the ones of Polα/Primase mutants in regard to template switch reduction and increased mutagenesis.^1^ These findings suggested that replicative-helicase coupled repriming, mediated in the context of the Ctf4/Polα/Primase complex, represents an innate response to transient fork stalling and an integral part of DDT (Fig. 1).

**Figure 1.**
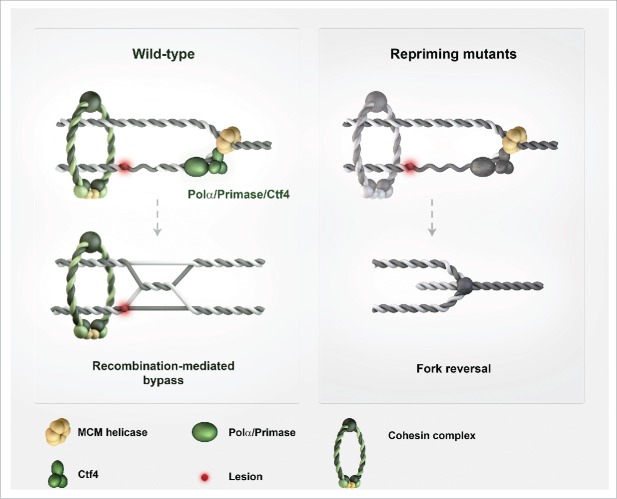
Effects of repriming on DNA damage tolerance (DDT), fork topology and sister chromatid cohesion. Efficient repriming supports postreplicative error-free DDT by template switching and sister chromatid cohesion. Defective replicative helicase-coupled repriming causes an increase in single-stranded (ss) DNA stretches at the fork and fork reversal. These events are accompanied by a shift in the location of DDT with respect to the replication fork, causing a different usage of DDT pathways. The observed negative effects on sister chromatid cohesion are likely the complex interplay between defective ssDNA metabolism and altered DDT and replication fork architecture. Defects in repriming and cohesion are graphically represented by the gray color of the complexes mediating these reactions.

Ctf4 also plays a role in sister chromatid cohesion.^15^ Cohesion is in large part mediated by cohesin, an evolutionarily conserved protein complex that structurally resembles a ring that holds sister chromatids together.^16^ Cohesin is loaded on chromosomes in G1, but the cohesive function of cohesin is only manifested in S phase, when cohesin is acetylated. How Ctf4 contributes to cohesion is not understood. Genetically, Ctf4 role in sister chromatid cohesion is manifested in collaboration with other replisome-associated cohesion factors,^17^ none of which are an integral part of the cohesin ring. Because cohesin acts as a splint to guide the repair of broken mitotic chromosomes toward the sister chromatid template,^18,19^ we reasoned that this function of cohesin may be shared by other cohesion factors, including Ctf4, and be relevant for other recombination-associated processes, such as template switching. In this new light, we wondered if the cohesion function of Ctf4, rather than the one of coupling repriming with the replicative helicase, was responsible for the observed template switch defects associated with *CTF4* deletion.^1^ Given the complexity in Ctf4 functions, we needed additional investigations to understand which of the above-mentioned roles of Ctf4, or both, were underlying the observed DDT defects of *ctf4* mutants.

We hypothesized that if cohesion were the important function of Ctf4 in facilitating template switching, affecting cohesion by diverse means should cause a similar DDT defect. Indeed, we found that mutations or conditional depletion of cohesin reduced the efficiency of template switch intermediate formation.^1^ We then asked if the observed template switch defects observed in Primase mutants were also in fact associated with cohesion defects – similarly to the case of Ctf4 –, or if the mechanisms by which Ctf4 and Polα/Primase facilitated template switching were distinct from each other. No roles for Polα/Primase in cohesion had been reported before, but direct interrogation of this issue revealed that this was indeed the case. Moreover, genetically, the cohesion defects of Ctf4 and Polα/Primase mutants appeared epistatic.^1^ Thus, Ctf4 and Polα/Primase act jointly to promote recombination-mediated DDT and cohesion (Fig. 1). However, was the relevant DDT function of Ctf4 and Polα/Primase the one in cohesion, the one of coupling replicative helicase with repriming, or both?

We proceeded to test the causal relationship between cohesion and template switching. For this purpose, we used a genetic trick that took into consideration the details of the LacO-LacI interaction module, previously used masterfully in a different context.^20^ With LacO arrays integrated at a known genomic location, we expressed either of two different forms of LacI: a wild-type form of LacI that can interact with LacO modules placed on both sister chromatids (the tetramer form), or a mutated version that can only interact with LacO arrays placed on a single chromatid (the dimer form). The tetramer form of LacI promotes local sister chromatid tethering acting locally as artificial cohesion; the dimer form of LacI does not. To test the potency of this system, we began by testing if the role of cohesin in template switching was due to its ability to keep the sister chromatids together. Indeed, the local template switch defect associated with cohesin mutants could be rescued by LacO-LacI mediated local sister chromatid tethering, but not by expressing the dimer form of LacI.^1^ Moreover, as expected, the employed artificial cohesion system could only rescue proximal template switch defects, and not the ones on other chromosomes.^1^ Thus, we could demonstrate that cohesin facilitates template switching by its structural role in mediating sister chromatid proximity.

We next addressed the role of artificial cohesion on the local template switch defects of Ctf4 and Primase mutants. However, differently from cohesin, the recombination defects associated with these mutations could not be rescued by inducing artificial cohesion, although the system was functional in inducing local sister chromatid proximity.^1^ Thus, the cohesion dysfunctions of Ctf4/Primase mutants were not the cause of the associated DDT defects. We concluded that Ctf4/Polα/Primase function enables robust cohesion and template switch during replication, by a mechanism fundamentally different from the one of cohesin. Moreover, while cohesion-mediated sister chromatid proximity supports recombination, in case of *ctf4* mutants, the cohesion and template switch defects were not linearly linked. Could it be that the template switch defects were underlying the cohesion defects, or that both of these problems were born from yet a different condition?

To approach these questions, we decided to use genetics and electron microscopy of genome-wide replication intermediates. Both approaches pointed to mismanagement of ssDNA at the fork: we observed longer stretches of ssDNA proximal to the fork junction, and an increase in fork reversal events in these mutants (Fig. 1), while no reversed forks were observed in control wild-type cells.^1^ Genetically, Ctf4/Primase mutants depended on viability on recombination factors with annealing activities, such as Rad52/Rad59, and on the ssDNA-binding activity of RPA. Moreover, in genetic tests, we found increased faulty annealing events to be highly elevated in Ctf4/Primase mutants.^1^ The simplest conjectural explanation is that defective replicative helicase-coupled repriming brings about increased fork remodeling associated with fork reversal, as well as faulty annealing events caused by the persistent ssDNA. This can well account for the reduction in postreplicative template switching and increased mutagenesis, while explaining the increased fork reversal we observed in Ctf4/Primase mutants (Fig. 1). However, future studies will be needed to interrogate the relationship between fork reversal and DDT.

The current model predicts that once repriming has occurred, defects in other key template switch factors would only cause increased TLS-mediated bypass rather than also increased fork reversal. On the contrary, in conditions of deficient repriming, alteration of other DDT factors that physiologically act primarily postreplicatively should cause a further increase in fork reversal. Examination of these predictions will likely bring forward a better understanding on the coordination of DDT with fork movement and remodeling, and will likely provide new handles to investigate the integration of these events within known DNA damage response circuits.

Perhaps even more challenging will be to reveal how DDT defects and fork topology alterations brought about by defective repriming negatively influence sister chromatid cohesion (Fig. 1). Is the sheer amount of persistent ssDNA, its location to the fork junction, or the complex effect of ssDNA mismanagement on fork topology and DDT that has the most important say in this issue? Can the DDT and cohesion defects of Ctf4/Primase mutants be uncoupled genetically? As sister chromatid cohesion is established during replication,^16,21^ and chromosome structural abnormalities and cohesion defects are also frequently observed in cancers,^22,23^ understanding the connections between DDT defects, fork topology, replication stress and sister chromatid cohesion perturbations appears both timely and highly relevant. With the continued pursuit of understanding replication stress, cohesion mechanisms, and how these two processes influence genome integrity, new insights are hopefully forthcoming.
